# Comparative Morphometric Study of the Occipital Condyle in Class III and Class I Skeletal Malocclusion Patients

**DOI:** 10.3390/diagnostics14232688

**Published:** 2024-11-28

**Authors:** Ismail Gumussoy, Suayip Burak Duman, Ozkan Miloglu, Mustafa Sami Demirsoy, Ayhan Dogan, Ahmed Z. Abdelkarim, Mustafa Taha Guller

**Affiliations:** 1 Department of Oral and Maxillofacial Radiology, Faculty of Dentistry, Sakarya University, 54100 Sakarya, Turkey; 2 Department of Dentomaxillofacial Radiology, Faculty of Dentistry, İnonu University, 44000 Malatya, Turkey; 3 Department of Dentomaxillofacial Radiology, Faculty of Dentistry, Ataturk University, 25030 Erzurum, Turkey; 4 Dentomaxillofacial Surgery, 54100 Sakarya, Turkey; 5 Orthodontics, 44100 Malatya, Turkey; 6 Department of Oral and Maxillofacial Radiology, Faculty of Dentistry, Ohio State University, Columbus, OH 43210, USA; 7 Department of Oral and Maxillofacial Radiology, Faculty of Dentistry, Giresun University, 28200 Giresun, Turkey

**Keywords:** occipital condyle, morphometric study, Class III malocclusion, cone beam computed tomography

## Abstract

Objectives: Since the formation of skeletal malocclusions is closely linked to general craniofacial development, it is crucial to understand the anatomy and growth patterns of the skull base. This study aimed to assess the morphometry of the occipital condyle (OC) on CBCT scans of Class III skeletal malocclusion subjects and compare the findings with those of skeletal Class I malocclusion subjects. Methods: A retrospective analysis was performed on CBCT images based on predefined inclusion and exclusion criteria. The sample consisted of 76 CBCT images of 38 skeletal Class III patients and 38 skeletal Class I patients. CBCT scans were used to measure mesiodistal width, sagittal length, coronal height, effective height of OC, and sagittal OC angle. Statistical analyses were conducted with RStudio software. Results: Significant differences were found in sagittal OC angle and sagittal length of OC between the study groups (*p* < 0.001). In other metrics, such as coronal height of OC, effective OC height, and mesiodistal width of OC between the groups, no significant differences were found. Class III malocclusions exhibited significantly reduced sagittal OC angle and sagittal length of OC compared to Class I malocclusions. The left side showed a significantly larger sagittal OC angle than the right side (*p* = 0.002). Conclusions: This preliminary study identified reduced sagittal angle and sagittal length of OC in patients with Class III skeletal malocclusion. Clinicians should recognize potential differences in OC morphometry in patients with skeletal malocclusions. Future studies involving larger populations are recommended to further investigate the relationship between skeletal malocclusions and posterior cranial base structures, including the OC.

## 1. Introduction

Angle’s initial method for classifying malocclusions, used in 1899, focused on the vertical alignment of the first molars and the alignment of the teeth in relation to the occlusal path [1]. However, it was later acknowledged that this classification, which relied on traditional theories of craniofacial development and growth, was inadequate. To enhance the comprehension of different skeletal occlusal patterns, it is essential to understand craniofacial growth and development, along with the function of the skull base [2,3]. The skull base plays a crucial role in the development of Class III skeletal facial patterns, as the integrity and growth of craniofacial structures directly influence the sagittal jaw relationships [4].

Brodie, in one of his studies [5], emphasized the importance of clinicians understanding the anatomy and growth patterns of the cranial base, as skeletal malocclusion development heavily relies on overall craniofacial growth. Therefore, clinicians should consider that the maxillofacial bone structures are intricately connected to the cranial base, and any alterations in the skull base may significantly impact the relationships between facial structures [6]. For instance, in cases of positional posterior plagiocephaly, defined as the deformity or flattening of the head due to persistent posture in neonates, the occipital bone is primarily affected. This asymmetric deformational process in the occipital bone, which occurs in the first months of life, affects both the skull base and the facial skeleton. A study by James et al. [7] analyzing the differences in the cranial base and facial skeleton of patients with deformational plagiocephaly showed that the external auditory meatus and the articular fossa were displaced anteriorly on the affected side in these patients.

Moyers [8] reported that the cranial base is one of the most stable regions of the skull, and its development and growth directly affect the alignment of facial structures such as the maxilla and mandible. Some authors have compared the skull base to a template for the development of the jawbones. Enlow [9,10] explained that an increase in the cranial base angle causes mandibular regression, while a decrease in the angle results in the mandible moving forward. In a study involving cephalometric radiography of patients with skeletal Class I and Class III malocclusions, Proff et al. [11] reported that a decreased cranial base angle was clearly associated with skeletal mandibular protrusion, and the results were consistent with the hypothesis of inadequate orthocephalization during Class III morphogenesis. A study by Marquez [12] stated that the anterior cranial base is smaller in individuals with mandibular protrusion. In another cephalometric study of the Southern Chinese population, Chin et al. [13] reported a significant correlation between the cranial base and jaw base relationship, with this correlation being particularly strong in skeletal Class III malocclusions. Additionally, Sanborn et al. [14] reported a smaller SN value in patients with Class III malocclusion compared to patients with Class I malocclusion.

The cranial base consists of several cranial bones, including the sphenoid, temporal, parietal, ethmoid, frontal, and occipital bones, which are connected by synchondroses [15]. The occipital bone, located in the posterior part of the cranial base, is a crucial component of the occipitocervical complex, particularly with the occipital condyles (OC). The OC plays a vital role in the craniovertebral junction (CVJ) by connecting the cranium to the vertebrae, thereby ensuring the structural stability and integrity of the CVJ [16,17].

Ensuring the stability of the CVJ is crucial for safeguarding the vital neurovascular structures within it. If the integrity of the CVJ is compromised, fusion and decompression of the occipitocervical complex are necessary to relieve pressure on these critical structures. Neoplastic diseases, hereditary or developmental anomalies, and traumas can lead to instability in the CVJ, requiring surgical fixation and fusion. OC-mediated fixation screws offer a viable option for stabilizing the CVJ [18,19]. However, owing to the complex anatomy and significant variations in the occipital condyle, various morphological measurements have been documented in skull analyses, cadaveric studies, and CT scans [20,21]. Additionally, the proximity of critical structures like the vertebral artery, jugular foramen, and hypoglossal canal to the OC underscores the importance of the CVJ and the challenges associated with surgical procedures [20,22].

Consequently, there are many studies in the literature regarding the posterior cranial base and skeletal Class III malocclusions. In one such study, Sanggarnjanavanich et al. [23] stated that the anterior cranial base relates to the position of the maxilla, whereas the posterior cranial base is associated with the positions of the glenoid fossa and the mandible. Most of these studies used the Sella (S) and Basion (Ba) as reference points for the posterior cranial base. Although many morphometric studies have been performed using CT scans to anatomically assess the OC [24,25,26], no studies have been found in the literature regarding the OC morphometry, which is a crucial component of the posterior cranial part and is in close proximity to the Basion point, in relation to skeletal Class III malocclusions. The objective of this study was to clarify whether there is a correlation between OC morphometry and skeletal Class III malocclusion by comparing it with skeletal Class I malocclusion. To achieve this aim, this paper conducts a morphometric assessment of the OC in subjects with Class III skeletal malocclusion using cone beam computed tomography (CBCT) and compares the findings with those of individuals with skeletal Class I malocclusion.

## 2. Materials and Methods

In this retrospective study, patients who were referred to Inönü University’s Faculty of Dentistry in Malatya, Türkiye, for orthodontic treatment were used. Ethical approval was obtained from the Health Sciences Ethics Committee of Inonu University (Reference date/no: 2024/6129). All processes involving human participants adhered to the 1964 Helsinki Declaration and its subsequent amendments.

The study’s sample size was determined using the G*Power software (version 3.1.9.7; Franz Faul, University of Kiel, Kiel, Germany). With an α error probability of 0.05 and a power of the study of 0,80, the actual power of the study was calculated to be 80% when at least 38 samples per group were included. The inclusion criteria specified an ANB angle under 0°, Class III molar occlusion, and negative overjet for patients with Class III malocclusion. The inclusion criteria specified an ANB angle ranging from 0° to 4°, Class I molar occlusion, and a normal overjet and overbite for Class I on available CBCT images. The exclusion criteria included a previous history of orthodontic treatment, craniofacial trauma or malformation, evidence or history of medical complications or syndromes, severe crowding, and insufficient scans of craniofacial structures.

The scans were performed using a CBCT machine (NewTom 5G, QR, Verona, Italy); 110 kVp, 1–11 mA, 3.6 s, 15 × 12 cm^2^ field of view (FOV) were obtained with 0.3 mm^3^ voxel size parameters. Post-reconstruction, DICOM files were transferred to RadiAnt image viewer (RadiAnt 4.0.2, Poznań, Poland). Metric and angular measurements were performed with RadiAnt software. All measurements were conducted by consensus of two maxillofacial radiologists (S.B.D. and M.T.G.), both of whom had at least 4 years of experience in maxillofacial radiology. To assess intraobserver reliability, the same radiologists repeated measurements after 15 days. The intraclass correlation coefficient (ICC) was calculated for all measurements, with values ranging between 0.88 and 0.86.

**Figure 1:**
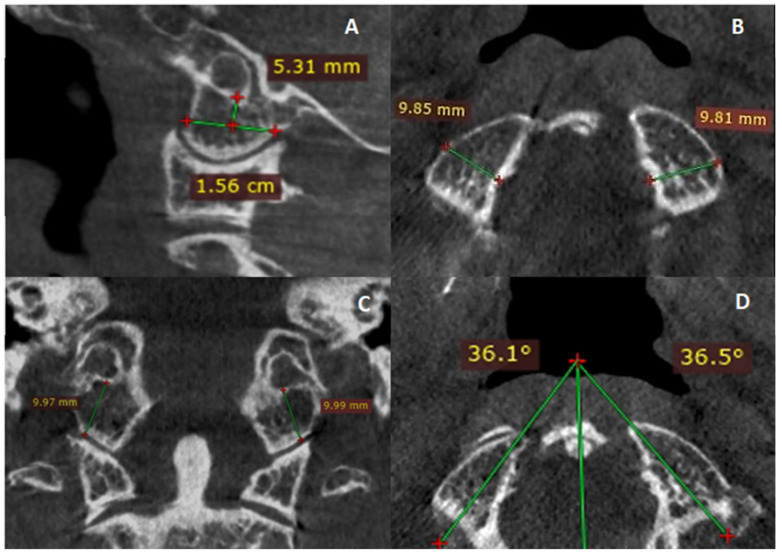
OC morphometry in different planar views. (**A**) Sagittal length and effective height of OC on sagittal slice. (**B**) Mesiodistal width of OC on axial slice. (**C**) Coronal height of OC. (**D**) Sagittal OC angle of the left and right OC on axial slice.

The mesiodistal width of OC (OC-MDW), sagittal length of OC (OC-SL), sagittal OC angle (OC-SA), coronal height of OC (OC-CH), and effective OC height (OC-EH) were measured (Figure 1).

OC-MDW, OC-SL, OC-SA, OC-CH, and OC-EH were measured based on the study by Gumussoy et al. [27]. OC-SL was measured alongside the long axis of OC on the parasagittal plane. OC-MDW was measured as the broadest line perpendicular to the midpoint of the long axis, extending from the lateral to medial margin on the horizontal plane. The extended line descending from the hypoglossal canal to the condylar cartilage in the frontal plane was measured as the OC-CH. The angle between the OC’s long axis and the sagittal midline was defined as OC-SA. OC-EH, necessary for OC screw passage with at least 3.5 mm diameter, was measured via the passageway above the occipitocervical joint and beneath the hypoglossal canal [20].

### Statistical Analysis

Descriptive statistics, including the basic characteristics of the study groups, were summarized using means and standard deviations. Sex distribution in each class was evaluated using percentages. There was homogeneity of variances between groups, as assessed by a Levene’s test for equality of variances, and parametric tests were conducted as the Shapiro–Wilk test confirmed that the variables followed a normal distribution. Independent samples *t*-tests were conducted to determine differences in quantitative variables (significance level set at *p* = 0.05). Paired *t*-tests were performed to analyze differences in occlusal parameters between the right and left sides. Statistical tests were performed with RStudio program (R software version: 4.2.2) to calculate *p*-values and assess statistical significance.

## 3. Results

The basic features of the study groups are given in Table 1. The sample consisted of 76 CBCT scans: 38 from skeletal Class III patients (ages 18 to 43) and 38 from skeletal Class I patients (ages 19 to 35).

Table 2 presents a comparison of OC measurements between skeletal Class III and Class I. Statistical analysis showed a significant difference in the OC-SL (*p* < 0.001). Especially, patients with skeletal Class III malocclusions showed a shorter OC-SL compared to patients with Class I. Furthermore, OC-SA showed significant differences between the study groups on both the right and left sides (*p* = 0.02, *p* = 0.005).

**Figure 2:**
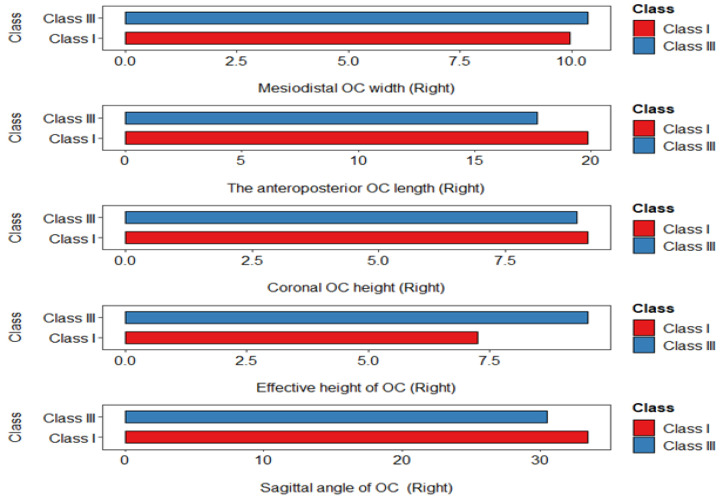
Figure exhibits the comparative table of OC measurements among study groups (right side).

**Figure 3:**
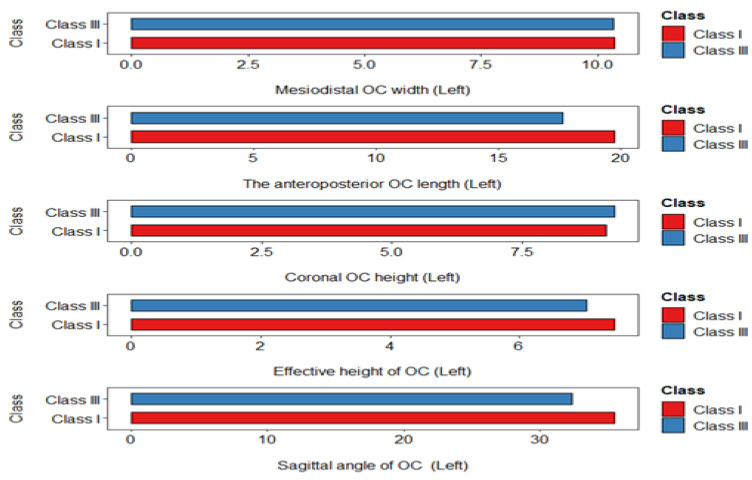
Figure exhibits the comparative table of OC measurements among study groups (left side).

Yet, there were no significant differences detected in OC-MDW, OC-CH, and OC-EH between the malocclusion groups (Figure 2 and Figure 3).

Table 3 outlines the differences among the genders in the overall study population and in each malocclusion group.

**Figure 4:**
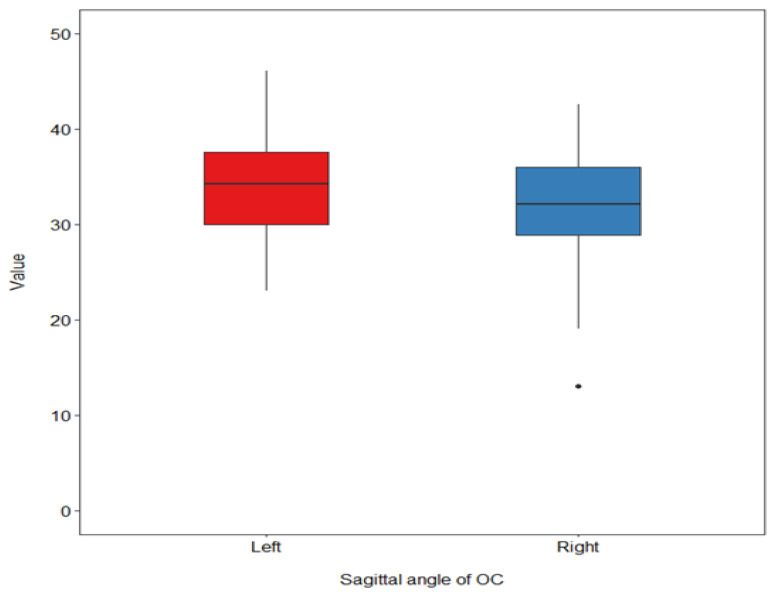
A box plot chart: comparison of OC-SA between the right and left condyle.

As shown in Table 4, no significant difference was detected in OC-MDW, OC-SL, OC-CH, and OC-EH between the right and left sides. However, OC-SA was greater on the left side at *p* = 0.002 (Figure 4).

## 4. Discussion

The current orthodontic perspective extends beyond just teeth and occlusion. Advances in scientific literature have deepened our comprehension of the link between craniofacial development and malocclusions, offering a holistic perspective on occlusion, temporomandibular function, and craniofacial structures. The cranial base has long piqued the curiosity of both clinicians and anthropologists. So far, various studies have focused on OC, a crucial element of the cranial base. Most studies have targeted OC morphology in certain populations or looked into implantation angles and directions for occipitocervical fixation surgeries. This study delves into the link between OC morphometry and skeletal Class III facial patterns, contrasting them with skeletal Class I individuals. The study found statistically significant differences in sagittal OC angle (OC-SA) and sagittal OC length (OC-SL) between the groups. (*p* = 0.00). No significant differences were found in the mesiodistal width of OC (OC-MDW), coronal height of OC (OC-CH), and effective OC height (OC-EH) between the groups.

In this study, the sagittal angle and length of the OC in Class III skeletal patients were significantly lower than those in Class I patients. When evaluating the correlation between OC morphology and skeletal malocclusion, there were several reasons for preferring only Class III cases in this study. The cranial base consists of both anterior and posterior parts. The anterior cranial base is associated with the position of the upper jaw, while the posterior cranial base is connected to the positions of the lower jaw and the glenoid fossa [23,28,29]. According to many studies in the literature, a smaller cranial base angle and shorter posterior cranial base length are major morphological characteristics of skeletal Class III patients [11,29,30,31,32]. Therefore, the anatomical proximity of the OC to the posterior cranial base, which ends with the Basion point, is an interesting aspect for Class III cases. Theisen et al. [4], in their study on the relationship between the cranial base and skeletal malocclusion, found a shorter posterior skull base length in subjects with skeletal Class III malocclusion. Enlow [9], in his study on the skull base and mandible relationship, observed that individuals with a shortened cranial base tended to have a more brachycephalic (broad and short) head shape. Additionally, Triwardhani et al. [33] and Moullas et al. [34] observed that individuals with a brachycephalic head type tend to have a wider, shorter, and more angular skull base, resulting in relative mandibular protrusion. These findings may support the results observed in the present study. Thus, patients with skeletal Class III malocclusion often have a brachycephalic head type, which suggests that their OC might be shorter. Furthermore, given the nature of the brachycephalic head shape, a decrease in the sagittal OC angle might be anticipated. Xue et al. [35] noted that genetics play a significant role in facial growth, with genetic effects being more pronounced in certain malocclusions, particularly skeletal Class III cases. In the present study, females exhibited a larger OC-MDW than males, across the entire population. Conversely, males showed a greater OC-SA compared to females, hinting at a possible sex-related difference in occlusal patterns. In the Class I malocclusion subgroup, females exhibited both a larger OC-MDW and a longer OC-SL compared to males. However, research on gender differences in OC is limited. Hormonal differences may contribute to these disparities, as testosterone is known to promote the development of larger bone volumes, unlike estrogen. Additionally, differences in the genetic encoding of the X and Y chromosomes, which influence bone deposition and bone mass, may also explain this variation [36]. 

Despite a wealth of literature on the link between Class III skeletal malocclusion and cranial base morphometry, the findings remain controversial. Many researchers have provided evidence that the skull base significantly affects the sagittal relationship of the jawbones [12,14,37,38,39,40]. Singh [41] observed that cranial base morphometry in patients with skeletal Class III malocclusion differed from that in patients with skeletal Class I malocclusion due to insufficient flattening or orthocephalization of the skull base during development. Moyers [8] highlighted that the growth of the cranial base directly impacts the vertical relationship between the jaws, while Enlow [9] described the cranial base as the template for facial development, asserting that changes at the cranial base directly impact the dimensions, alignment, and angles of maxillofacial structures. In a study exploring the relationship between the skull base and mandible in patients with both mandibular prognathism and facial asymmetry, Kim et al. [42] reported that cranial base and mandibular volumes increased on the non-deviated side, with a significant correlation between them. They emphasized the importance of considering cranial base asymmetry when planning treatment for patients with mandibular prognathism and facial asymmetry. Kluba et al. [43] found an increased prevalence of malocclusions in patients with positional plagiocephaly compared to control subjects. The rise in malocclusions in plagiocephaly cases, where asymmetric deformities in the posterior cranial structures occur due to persistent posture in infants, underscores the close relationship between facial and cranial bone structures.

Despite the existence of studies suggesting that skull base morphometry is a factor in the development of malocclusions, some researchers contend that the evidence supporting this claim is lacking. In a systematic review, Almeida et al. [44] emphasized that the cranial base angle isn’t crucial for the development of malocclusions. Similarly, Dhopatkar et al. [45] concluded that the cranial base angle was not fundamentally significant in explaining malocclusion, given the considerable variation in mandibular morphology across different types of malocclusions. This paper investigated the relevance between OC morphometry, a crucial component of the cranial base, and Class III skeletal malocclusions. On the other hand, it is critical to consider that the morphogenetic facial structure comprises of multiple specific features unique to each face type, and no single feature can fully describe a facial model. Compensation mechanisms may also occur within maxillofacial structures, potentially mitigating abnormal skull base morphology. For example, in a study assessing mandibular asymmetry in patients with occipital bone asymmetry due to plagiocephaly, Baumler et al. [46] found that the asymmetry in the occipital bone was often not reflected in the mandible due to compensatory mechanisms. These determinants might clarify the conflicting results across various studies. Additionally, even in morphometric studies on overall population, OC measurements can differ among races, leading to varied outcomes across different radiologic techniques (e.g., cephalometric radiographs, CT, and CBCT). To our knowledge, no study has directly examined the relationship between skeletal malocclusion and OC morphometry, leaving a gap in the literature for direct comparison with our findings.

Despite our best efforts, the present study has a few limitations. First, data were collected from a single institution using a convenience sampling method, which limited the population diversity. Secondly, the sample size of this study was limited to 38 participants. While the use of CBCT ensures highly accurate and realistic measurements, it also results in a smaller sample size compared to studies using lateral cephalography, particularly in specific populations such as skeletal Class III malocclusion patients. Many studies comparing posterior cranial base morphometry with skeletal malocclusions in the literature were performed using lateral cephalography, which does not allow for the evaluation of OC morphometry [4,13,14,23,40,41]. Therefore, future research should employ larger sample sizes and explore varying skeletal malocclusions using different cephalometric methods to provide a more comprehensive understanding of this topic.

OC morphometry is also highly relevant in cranial and spinal surgeries [20,47]. In cases of occipitocervical complex instability, understanding OC morphology becomes critical for clinicians, especially in patients with craniofacial abnormalities such as skeletal Class III brachycephalic patterns. In Class III patients, this study noted a reduced OC angle and sagittal OC length, which may have important implications for surgical interventions involving CVJ instability. Since skeletal malocclusion patients can exhibit varying skull base morphometries, surgeons need to be well-informed about these differences to optimize treatment strategies.

## 5. Conclusions

This study evaluated OC morphometry in patients with Class III skeletal malocclusion and compared it with skeletal Class I to investigate a possible relationship between skeletal malocclusions and OC morphometry. It was found that the sagittal angle and sagittal length of the OC in Class III skeletal patients were significantly lower than those in Class I patients. No significant differences were found in the OC-MDW, OC-CH, and OC-EH between the study groups. The findings provide promising insights into a potential correlation between OC morphometry and skeletal mandibular prognathism. Future CBCT studies with a larger population are strongly recommended to better elucidate the relationship between Class III skeletal malocclusions and posterior cranial base structures such as OC.

## Figures and Tables

**Table 1:** Age and sex distribution of patients between study groups.

Variable	Level	Class I *n* = 38	Class III *n* = 38	*p*
Gender (%)	Female	19 (50.0)	19 (50.0)	1
	Male	19 (50.0)	19 (50.0)	
Age (mean (SD))		22.45 (3.77)	22.32 (5.08)	0.898

All continuous data arerepresented as mean standard deviations (SD). *p*-values for continuous data are derived from independent sample *t*-tests. *p*-values for categorical data were derived from the chi-square test.

**Table 2:** Comparison of the occipital condyle (OC) parameters between Class I and Class III malocclusions.

	Class I	Class III	Mean Difference	*p*-Value
Right				
OC-MDW	9.96 (1.30)	10.36 (1.32)	−0.4	0.190
OC-SL	19.84 (2.07)	17.67 (2.48)	2.17	<0.001
OC-CH	9.12 (1.40)	8.91 (1.81)	0.21	0.570
OC-EH	7.25 (1.63)	9.51 (12.05)	−2.26	0.250
OC-SA	33.42 (4.61)	30.47 (6.10)	2.95	0.020
Left				
OC-MDW	10.35 (1.43)	10.33 (1.21)	0.02	0.940
OC-SL	19.74 (1.94)	17.65 (2.93)	2.08	<0.001
OC-CH	9.11 (1.09)	9.27 (1.98)	−0.16	0.670
OC-EH	7.46 (1.60)	7.04 (2.01)	0.43	0.300
OC-SA	35.44 (4.43)	32.32 (5.01)	3.12	0.005

All continuous data are represented as mean standard deviations (SD). *p*-values for continuous data were derived from independent sample *t*-tests.

**Table 3:** Contrasting the genders within the different subgroups.

Overall			
Variable	Male *n* = 38	Female *n* = 38	*p*-Value
Right			
OC-MDW	9.86 (1.23)	10.45 (1.35)	0.048
OC-SL	18.31 (1.91)	19.21 (2.96)	0.120
OC-CH	8.99 (1.68)	9.04 (1.56)	0.904
OC-EH	8.96 (12.09)	7.80 (1.86)	0.560
OC-SA	33.23 (4.79)	30.66 (6.05)	0.043
Left			
OC-MDW	10.07 (1.21)	10.61 (1.37)	0.075
OC-SL	18.30 (2.36)	19.09 (2.95)	0.203
OC-CH	9.14 (1.83)	9.24 (1.32)	0.792
OC-EH	6.94 (1.93)	7.56 (1.66)	0.139
OC-SA	34.37 (4.91)	33.38 (5.02)	0.390
Class I			
Variable	Male *n* = 19	Female *n* = 19	*p*-value
Right			
OC-MDW	9.44 (0.97)	10.48 (1.40)	0.011
OC-SL	19.09 (1.75)	20.59 (2.13)	0.024
OC-CH	9.04 (1.32)	9.20 (1.51)	0.720
OC-EH	6.75 (1.55)	7.75 (1.60)	0.057
OC-SA	33.78 (4.30)	33.06 (4.99)	0.634
Left			
OC-MDW	9.95 (1.23)	10.75 (1.53)	0.083
OC-SL	19.22 (1.58)	20.26 (2.16)	0.100
OC-CH	9.03 (1.09)	9.18 (1.11)	0.674
OC-EH	7.55 (1.61)	7.37 (1.63)	0.740
OC-SA	36.47 (3.48)	34.40 (5.10)	0.152
Class III			
Variable	Male *n* = 19	Female *n* = 19	*p*-Value
Right			
OC-MDW	10.28 (1.34)	10.43 (1.34)	0.735
OC-SL	17.52 (1.78)	17.82 (3.07)	0.710
OC-CH	8.94 (2.01)	8.87 (1.64)	0.899
OC-EH	11.17 (16.97)	7.85 (2.13)	0.402
OC-SA	32.68 (5.29)	28.26 (6.17)	0.023
Left			
OC-MDW	10.19 (1.22)	10.46 (1.22)	0.499
OC-SL	17.38 (2.67)	17.92 (3.21)	0.579
OC-CH	9.24 (2.38)	9.29 (1.54)	0.947
OC-EH	6.33 (2.07)	7.74 (1.71)	0.028
OC-SA	32.26 (5.29)	32.37 (4.87)	0.949

All continuous data are represented as mean standard deviations (SD). *p*-values for continuous data were derived from independent sample *t*-tests. *p*-values for categorical data were derived from the chi-square test.

**Table 4:** Presents the comparative table of OC measurements among the right and left sides.

Variable	Right	Left	*p*-Value
OC-MDW	10.16 (1.32)	10.34 (1.32)	0.195
OC-SL	18.76 (2.52)	18.70 (2.68)	0.817
OC-CH	9.01 (1.61)	9.19 (1.59)	0.185
OC-EH	8.38 (8.61)	7.25 (1.81)	0.247
OC-SA	31.95 (5.57)	33.88 (4.96)	0.002

All continuous data are represented as mean standard deviations (SD). *p*-values for continuous data were derived from paired *t*-test. *p*-values for categorical data were derived from the chi-square test.

## Data Availability

The original contributions presented in this study are included in the article. Further inquiries can be directed to the corresponding author.
